# Selective use of grafted TIP urethroplasty in distal to proximal penile hypospadias: a single-center cohort study

**DOI:** 10.1007/s00345-026-06690-w

**Published:** 2026-08-21

**Authors:** P. D. T. Abazari, A. M. Schmidt, M. Stehr, F. M. Schäfer

**Affiliations:** 1 Department of Paediatric Surgery and Paediatric Urology, Diakoneo, Cnopfsche Kinderklinik, Nürnberg, Germany; 2 Department of Pediatric Surgery and Pediatric Traumatology, Surgical Center for Children (Operatives Kinderzentrum, OKiZ), Hospital St. Hedwig of the Order of St. John of God, University Children’s Hospital Regensburg (KUNO), Regensburg, Germany

**Keywords:** Hypospadias, Tubularized incised plate, Grafted tubularized incised plate, Snodgraft, Dorsal inlay graft urethroplasty

## Abstract

**Purpose:**

To compare standard tubularized incised plate urethroplasty (TIP) and selectively applied grafted TIP (GTIP) in primary single-stage penile hypospadias repair.

**Methods:**

We retrospectively analyzed boys undergoing primary, single-stage TIP or GTIP for distal or midshaft-to-proximal penile hypospadias repair at a tertiary pediatric center between 2012 and 2022. TIP was the default technique. GTIP was reserved for urethral plates < 8 mm, atrophic plates, or plates not safely tubularizable over an 8 Ch catheter without deep incision into the corpus spongiosum. Patients with follow-up ≥ 6 months were included. Primary outcomes were postoperative complications and revision surgery. Clinically relevant ventral curvature requiring correction was assessed as a potential confounder.

**Results:**

Overall, 146 patients were included, 107 undergoing TIP and 39 GTIP, with a mean follow-up of 37.2 months. Using the institutional intraoperative decision strategy, GTIP was reserved for unfavorable urethral plate characteristics or unfavorable tubularization conditions and was more frequently used in midshaft-to-proximal penile cases. Clinically relevant ventral curvature was observed in 20.6% of TIP and 17.9% of GTIP cases (*p* = 0.82). Observed complication rates were 34.6% after TIP and 33.3% after GTIP (37/107 vs. 13/39; RR 1.04, 95% CI 0.64–1.78), while revision rates were 29.0% and 30.8%, respectively (31/107 vs. 12/39; RR 0.94, 95% CI 0.56–1.68), without evidence of a clear between-group difference for either outcome. After adjustment for meatal location and curvature, estimates for surgical technique remained close to unity for both complications and revision. GTIP procedures had longer operative times and stent duration.

**Conclusion:**

Selectively applied GTIP in unfavorable cases yielded complication and revision rates closely aligned with those after TIP, supporting its use as a urethral-plate-guided adjunct when safe tension-free tubularization cannot otherwise be achieved.

**Supplementary Information:**

The online version contains supplementary material available at 10.1007/s00345-026-06690-w.

## Introduction

Hypospadias is among the most common congenital anomalies of the male external genitalia, with reported prevalence varying substantially between surveillance systems and populations [1, 2]. Most cases are distal or penile shaft variants and are amenable to single-stage repair. Over the last three decades, tubularized incised plate urethroplasty (TIP), described by Snodgrass, has become the standard technique for primary distal and selected penile shaft repairs because of its reproducibility, cosmetic acceptability, and generally favorable complication profile [3–5].

Despite its broad adoption, TIP has limitations in unfavorable urethral plates. Plate morphology is not defined by width alone, but also by groove depth, tissue quality, spongiosal support, and feasibility of tension-free tubularization. Although early studies linked narrow plates, often < 8 mm, to fistula formation and shallow grooves to stenosis, later studies questioned the predictive value of width as an isolated parameter. In practice, grafting is therefore often based on a composite intraoperative assessment rather than a single measurement [6–9].

To mitigate these risks, graft augmentation of the incised plate using preputial skin, termed grafted tubularized incised plate, grafted TIP, GTIP, Dorsal inlay graft urethroplasty (DIGU) or “Snodgraft“, was popularized as a way to epithelialize the dorsal incision and potentially reduce contracture [10–16].

Comparative evidence for TIP versus GTIP remains heterogeneous. Randomized and prospective studies in distal phenotypes have reported either comparable outcomes or reduced stenosis after grafting, usually at the cost of longer operative time [14, 16–18]. Meta-analyses suggest that grafting may reduce meatal or neourethral stenosis, whereas fistula and reoperation rates are less consistently affected [15, 19].

Two important gaps remain in the current literature. First, few comparative series report outcomes based on a clearly defined intraoperative decision algorithm for graft augmentation. Second, most studies inadequately distinguish between different anatomical severity features, including meatal location, ventral curvature, glans configuration, and urethral plate morphology. This distinction is important because grafted TIP is usually selected for urethral-plate-specific unfavorable anatomy rather than for curvature-related severity alone, introducing potential confounding by indication that is inconsistently captured in existing studies [8, 15, 18–20].

Therefore, the aim of this study was to evaluate outcomes of standard TIP and selectively applied GTIP in a single-center pediatric cohort using a consistent intraoperative decision strategy. We hypothesized that GTIP, when used for unfavorable urethral plate characteristics or unfavorable tubularization conditions, would not be associated with higher complication or revision rates compared with standard TIP.

## Materials and methods

### Study design and setting

This retrospective single-center study was conducted at the Cnopfsche Kinderklinik, Nuremberg, Germany, covering January 2012 to December 2022. Institutional ethics approval was obtained prior to data analysis (Ref. No. 19–198-Br, Friedrich-Alexander-Universität Erlangen (FAU)).

#### Eligibility criteria and cohort definition

All boys undergoing hypospadias surgery during the study period were screened. The analytic cohort included consecutive patients who underwent primary, single-stage TIP or GTIP for coronal, subcoronal, distal penile, midshaft, or proximal penile hypospadias and had follow-up ≥ 6 months. Redo procedures, hypospadias variants such as megalomeatus and procedures other than TIP or GTIP were not eligible. In line with commonly used classifications, hypospadias was categorized according to the preoperative meatal location. Distal hypospadias (“Distal”) comprised coronal, subcoronal, and distal penile forms, whereas the midshaft/proximal penile group (“Mid+Prox penile”) included midshaft and proximal penile forms [16–18]. The study size was determined by the number of consecutive eligible patients treated during the predefined study period. No formal sample size calculation was performed due to the retrospective design.

#### Surgical technique and decision algorithm

The preferred technique was classical TIP. Intraoperative assessment of the urethral plate guided the use of graft augmentation.

After penile degloving, an artificial erection test was performed without tourniquet to assess residual ventral curvature. According to institutional practice, residual curvature was corrected before urethroplasty when considered clinically relevant, corresponding to an institutional threshold of ≥ 20°. The exact method of angular quantification and the individual curvature degree were not consistently documented in the retrospective records. Correction was performed predominantly with plication sutures; in a small minority, multiple superficial transverse ventral corporotomies (“fairy cuts”) were used while in all cases preserving the urethral plate [21].

**Fig. 1:**
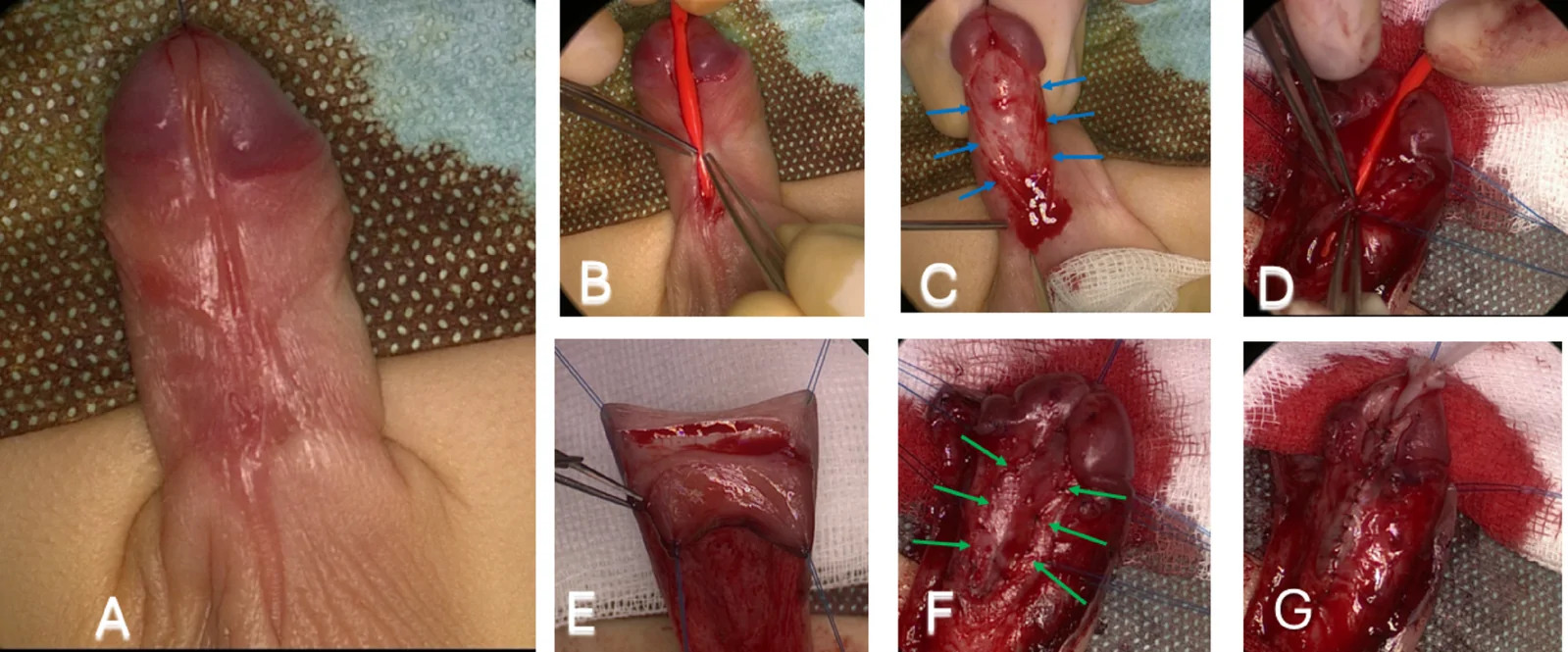
Intraoperative decision algorithm for urethral plate grafting and operative technique (illustrated). **a** Intraoperative appearance of an atrophic urethral plate **b** Tension-free tubularization over an 8-Ch catheter not feasible without deepening the incision. **c** Deep midline dorsal incision of the urethral plate into the corpus spongiosum, reaching the tunica albuginea; wound margins marked by light blue arrows. **d** Tension-free approximation of the urethral plate margins over an 8-Ch catheter after deep incision. **e** Harvesting of a free inner preputial skin graft. **f** Dorsal inlay graft sutured to the incised urethral plate; fixation sutures marked by green arrows. **g** Final tubularization over an 8-Ch catheter (Koyle diaper stent)

Following curvature correction, the urethral plate was evaluated. If tension-free tubularization over an 8 Ch catheter was feasible after a midline dorsal incision limited to the urethral plate, standard TIP was performed. In contrast, when the plate appeared narrow (approximately < 8 mm), atrophic, or could only be tubularized following a deep incision into the corpus spongiosum, the raw surface was covered with a dorsal inlay of inner preputial skin prior to tubularization (grafted TIP). The intraoperative decision algorithm and surgical technique are illustrated in Fig. 1. This intraoperative decision was based on surgeon judgment and reflects a pragmatic selection strategy rather than predefined objective measurements.

After creation of the neourethra, a second dartos layer was placed as a vascularized coverage layer in both groups. Glans wings were mobilized to allow tension-free glanuloplasty.

All procedures were performed by experienced pediatric urologists within a single institutional protocol. The intraoperative decision-making was consistently applied by two senior surgeons (MS and FMS) throughout the study period.

A urethral stent of 6 to 8 Ch (Koyle Diaper Stent, Cook Medical, USA) was typically left in place for 7 to 14 days.

### Outcomes and follow-up

The primary outcome was any clinically documented postoperative complication. Categories included meatal stenosis, urethrocutaneous fistula, glans dehiscence, infection, urethral breakdown, and other postoperative adverse events, independent of whether surgery was required. Meatal stenosis was defined as clinically relevant narrowing of the neomeatus diagnosed during follow-up and requiring operative correction. Outcomes were assessed during routine clinical follow-up by the treating pediatric urologists and extracted retrospectively from the medical records; no blinded or independent outcome adjudication was performed. Cosmetic appearance and voiding function were assessed clinically as part of routine care, but validated cosmetic scores and routine uroflowmetry were not available. Secondary outcomes included revision surgery and operative time. Follow-up visits were scheduled at catheter removal after 7 to 14 days, 3 months, 6 months, and annually thereafter. Only patients with follow-up of at least 6 months were included in the analyses.

For the primary outcome analysis, each patient was counted once as having a complication, regardless of the number of procedures required to treat the same underlying event.

For temporal analyses, complications were additionally recorded according to their time of occurrence. Patients could therefore be represented in more than one follow-up interval if distinct events occurred at different time points.

### Statistical analysis

Continuous variables are reported as mean ± standard deviation and median with interquartile range, as appropriate. Between-group comparisons were performed using the Mann–Whitney U test. Categorical variables were compared using Fisher’s exact test. Relative risks (RR) with 95% confidence intervals (CI) were calculated using the Koopman asymptotic score method.

Additionally, multivariable logistic regression analysis was performed to assess the association between surgical technique and outcomes while adjusting for potential measured confounders, including clinically relevant ventral curvature requiring correction (institutional threshold ≥ 20°) and meatal location. This analysis was exploratory and intended to evaluate whether the association between technique and outcomes was materially influenced by these documented anatomical factors.

A two-sided p-value < 0.05 was considered statistically significant. Missing data were not imputed. Analyses were performed using available documented data. Patients with follow-up below 6 months were excluded from all outcome analyses. Statistical analyses were performed using GraphPad Prism (version 10.6.1; GraphPad Software, San Diego, CA, USA).

## Results

### Cohort and follow-up

**Fig. 2:**
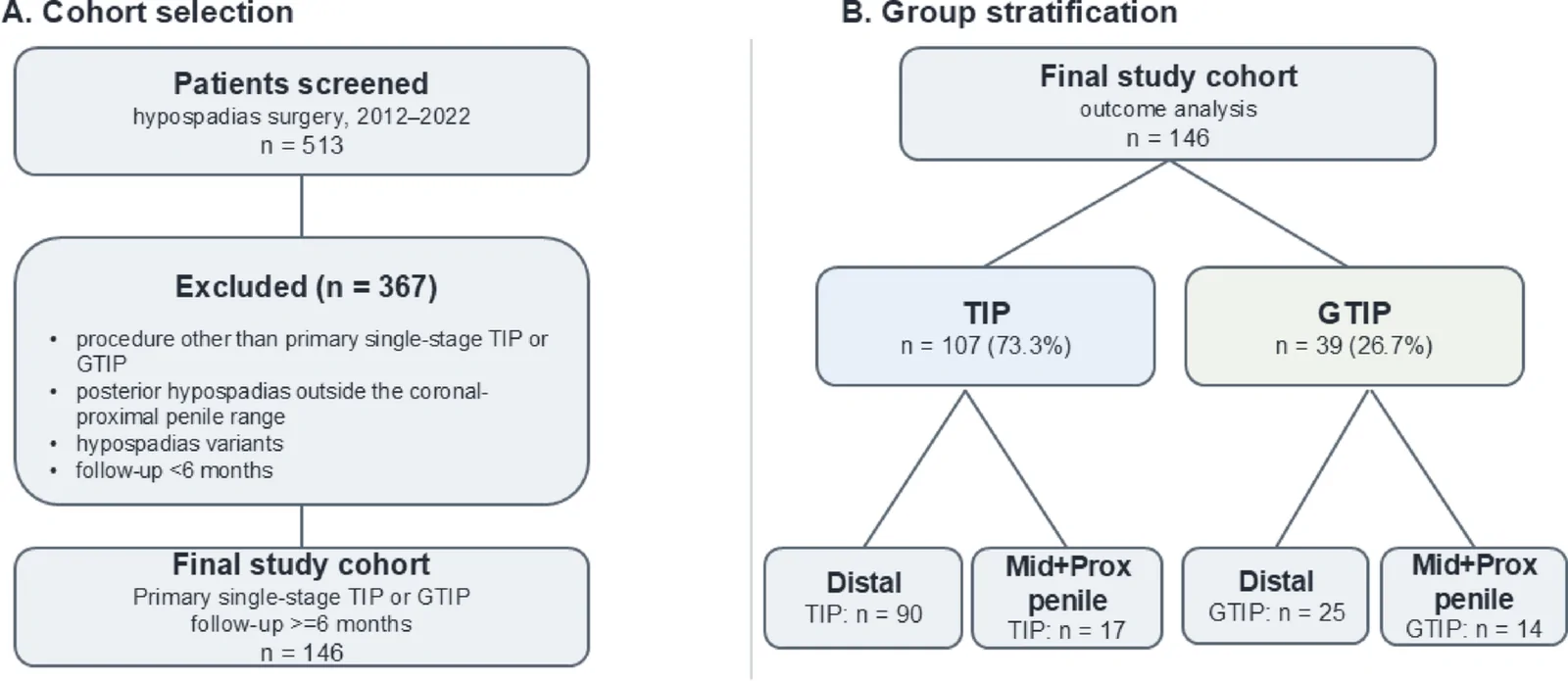
Study cohort selection and group stratification. Flow diagram of cohort selection and stratification by surgical technique and hypospadias localization. *TIP* Tubularized incised plate urethroplasty, *GTIP* Grafted tubularized incised plate urethroplasty. Distal hypospadias comprised coronal, subcoronal and distal penile forms; mid/proximal penile hypospadias comprised midshaft and proximal penile forms

Of 513 children screened, 146 met the inclusion criteria and had at least 6 months of follow-up. Standard TIP was performed in 107 patients (73.3%) and grafted TIP (GTIP) in 39 patients (26.7%). Cohort selection and stratification by surgical technique and hypospadias localization are shown in Fig. 2. Baseline characteristics are summarized in Table 1.

**Table 1:** Patient demographics, anatomical characteristics and perioperative parameters

Measure	Overall (n = 146)	TIP (n = 107)	GTIP (n = 39)	p-value
Age (months)	13.0 [10.9–16.7]	12.9 [11.0–16.8]	13.0 [10.7–16.7]	0.77
OT (min)	100 [85.8–118.5]	93.0 [82.0–106.0]	129 [115–136]	< 0.0001
Stented time (days)	10 [9–14]	10 [7–10]	14 [14–14]	< 0.0001
LOS (days)	2 [2–2]	2 [2–2]	2 [2–2]	0.27
Distal hypospadias, n (%)	115 (78.8)	90 (84.1)	25 (64.1)	0.01
Mid + proximal penile hypospadias, n (%)	31 (21.2)	17 (15.9)	14 (35.9)	0.01
Clinically relevant curvature (≥ 20°), n (%)	29 (19.9)	22 (20.6)	7 (17.9)	0.82

Data are presented as median [interquartile range] or n (%). Continuous variables were compared using the Mann–Whitney U test; categorical variables were compared using Fisher’s exact test. Clinically relevant ventral curvature requiring correction was assessed after degloving and artificial erection testing and corresponded to the institutional correction threshold of ≥20°.

*OT* Operative time, *LOS* Length of hospital stay, *IQR* Interquartile range

The mean age at surgery was 16.3 months (median 13.0), with a mean follow-up of 37.2 months. Median follow-up was 26.4 months [14.2–54.3] after TIP and 32.2 months [13.8–53.2] after GTIP. Detailed follow-up distribution by surgical technique and localization is provided in Supplementary Table 1.

Distal hypospadias predominated, while approximately one fifth were classified as midshaft to proximal penile.

The distribution of surgical techniques differed significantly by localization. In distal hypospadias, standard TIP was used in 78.3% (90/115) and GTIP in 21.7% (25/115). In contrast, GTIP was more frequently applied in midshaft to proximal penile cases (45.2%, 14/31), compared to standard TIP (54.8%, 17/31; Fisher’s exact test, *p* = 0.01).

### Operative parameters

Operative time was significantly longer in the GTIP group (median 129 vs. 93 min). Similarly, stent duration was prolonged (median 14 vs. 10 days). Age at surgery and length of hospital stay did not differ between groups (Table 1).

### Ventral curvature and intraoperative management

Residual ventral curvature was assessed intraoperatively using an artificial erection test. According to institutional practice, correction was undertaken only when curvature was considered clinically relevant, corresponding to the institutional threshold of ≥ 20°; exact individual angles were not consistently available retrospectively. Clinically relevant ventral curvature was observed in 29 of 146 patients (19.9%). It was present in 22 of 107 patients in the TIP group (20.6%) and in 7 of 39 patients in the GTIP group (17.9%), with no statistically significant between-group difference (Table 1). Thus, relevant ventral curvature showed no measurable imbalance between techniques. Correction was performed with plication sutures in 27 patients and by multiple superficial transverse ventral corporotomies (“fairy cuts”) in two patients. The urethral plate was preserved in all cases, and single-stage TIP or GTIP was subsequently completed.

### Complications

At least one complication occurred in 50 of 146 children (34.2%): 37 of 107 after TIP (34.6%) and 13 of 39 after GTIP (33.3%; RR 1.04, 95% CI 0.64–1.78; *p* > 0.99). Fistula was the most common complication (TIP 22/107 [20.6%] vs. GTIP 5/39 [12.8%]), followed by meatal stenosis (10/107 [9.4%] vs. 4/39 [10.3%]) and glans dehiscence (5/107 [4.7%] vs. 3/39 [7.7%]). Other complications included residual prepuce/phimosis, buried penis features, postoperative bleeding or hematoma, and constricting preputial rings.

No statistically significant differences were observed between techniques. Detailed estimates including relative risks and p values are provided in Table 2.

**Table 2:** Postoperative outcomes after TIP vs. GTIP

Outcome	TIP n (%)	GTIP n (%)	RR [95% CI]	p-value
*Overall cohort (n = 146, TIP 107, GTIP 39)*				
Any complication (≥ 1)	37 (34.6)	13 (33.3)	1.04 [0.64–1.78]	> 0.99
Meatal stenosis	10 (9.4)	4 (10.3)	0.91 [0.33–2.66]	> 0.99
Fistula	22 (20.6)	5 (12.8)	1.60 [0.70–3.93]	0.34
Glans dehiscence	5 (4.7)	3 (7.7)	0.61 [0.17–2.24]	0.44
Other (minor/infection)	4 (3.7)	4 (10.3)	0.36 [0.10–1.30]	0.21
Any revision (≥ 1)	31 (29.0)	12 (30.8)	0.94 [0.56–1.68]	0.84
*Distal hypospadias (n = 115, TIP 90, GTIP 25)*				
Any complication (≥ 1)	32 (35.6)	8 (32.0)	1.11 [0.62–2.18]	0.82
Meatal stenosis	9 (10.0)	2 (8.0)	1.25 [0.34–5.01]	> 0.99
Fistula	18 (20.0)	3 (12.0)	1.67 [0.60–5.11]	0.56
Glans dehiscence	4 (4.4)	3 (12.0)	0.37 [0.10–1.43]	0.17
Other (minor/infection)	4 (4.4)	3 (12.0)	0.37 [0.10–1.43]	0.17
Any revision (≥ 1)	27 (30.0)	7 (28.0)	1.07 [0.57–2.23]	> 0.99
*Midshaft + proximal penile hypospadias (n = 31, TIP 17, GTIP 14)*				
Any complication (≥ 1)	5 (29.4)	5 (35.7)	0.82 [0.31–2.24]	> 0.99
Meatal stenosis	1 (5.9)	2 (14.3)	0.41 [0.06–2.89]	0.58
Fistula	4 (23.5)	2 (14.3)	1.65 [0.41–7.05]	0.66
Glans dehiscence	1 (5.9)	0 (0.0)	Not estimable	> 0.99
Other (minor/infection)	0 (0.0)	1 (7.1)	Not estimable	0.45
Any revision (≥ 1)	4 (23.5)	5 (35.7)	0.66 [0.22–1.92]	0.69

Data are presented as n (% within treatment group). Relative risks and 95% confidence intervals were calculated using the Koopman asymptotic score method and are expressed as TIP versus GTIP. P values were calculated using Fisher’s exact test. “Any complication” and “Any revision” indicate the occurrence of at least one event.

*TIP* Tubularized incised plate urethroplasty, *GTIP* Grafted tubularized incised plate urethroplasty, *RR* Relative risk, *CI* Confidence interval

Subgroup analyses stratified by curvature showed no significant differences between techniques in patients with clinically relevant curvature (*p* = 0.15) or without such curvature (*p* = 0.67).

### Revision surgery

Reoperation was required in 43 of 146 children (29.5%): 31 of 107 after TIP (29.0%) and 12 of 39 after GTIP (30.8%; RR 0.94, 95% CI 0.56–1.68; *p* = 0.84). No relevant differences were observed across localizations. Detailed stratified outcome data are provided in Table 2.

### Multivariable analysis

In multivariable logistic regression adjusting for meatal location and clinically relevant ventral curvature.

requiring correction, surgical technique was not independently associated with complications (OR 1.12, 95% CI 0.50–2.45) or revision surgery (OR 1.07, 95% CI 0.46–2.42). Neither relevant ventral curvature nor localization was significantly associated with either outcome.

### Temporal distribution of complications

Complications were recorded across all follow-up intervals. Within the first 24 months, 24 patients had at least one complication. Among the 29 patients with follow-up ≥ 60 months, 10 had at least one complication recorded after 60 months (34.5%). Because long-term follow-up was selective and patients could contribute to more than one interval, these interval-specific counts are descriptive and should not be interpreted as time-dependent or cumulative incidence estimates. The temporal distribution of complications is detailed in Supplementary Table 2.

## Discussion

In this single-center cohort of 146 children undergoing primary distal to proximal penile hypospadias repair, standard TIP and selectively applied GTIP showed closely aligned observed complication and revision rates, despite GTIP being reserved for less favorable urethral plate characteristics. GTIP was used according to a predefined intraoperative algorithm for unfavorable urethral plate morphology or unfavorable tubularization conditions. Relevant ventral curvature showed no statistically significant imbalance between groups and was corrected before urethroplasty, making curvature-related severity an unlikely explanation for the closely aligned observed rates.

Surgical technique was not independently associated with complications or revision after adjustment for meatal location and curvature. However, these findings should not be interpreted as proof of equivalence or superiority. Allocation was non-randomized, the groups were markedly unequal in size (TIP *n* = 107; GTIP *n* = 39), and GTIP was intentionally reserved for more challenging urethral plates. The confidence intervals include potentially clinically relevant effects, and a type II error due to the limited GTIP cohort cannot be excluded. GTIP was associated with longer operative time and prolonged stenting, reflecting the additional procedural steps required for graft harvesting and fixation.

### Comparative evidence and inclusion logic

Comparative studies of TIP and GTIP are difficult to interpret because inclusion criteria and indication strategies vary substantially. In unselected distal cohorts, randomized trials generally found comparable outcomes, apart from longer operative time with GTIP [14, 16]. Mouravas et al. reported fewer complications after grafting in a broader anatomical spectrum, particularly regarding stenosis [17]. In contrast, studies focusing on narrow urethral plates, including Eldeeb et al., did not show a clear advantage of grafting [18].

Meta-analyses reflect this variability: Silay et al. reported reduced meatal or neourethral stenosis after grafting, whereas Alshafei et al. found no statistically significant overall differences between techniques [15, 19]. These discrepancies likely reflect differences in patient selection, urethral plate characteristics, and confounding by indication. In this context, our cohort contributes real-world data from a selective urethral-plate-guided algorithm rather than routine grafting in anatomically unselected cases.

### Anatomical complexity

Anatomical severity in hypospadias is multidimensional and cannot be reduced to ventral curvature alone. Curvature is a recognized severity marker and has been associated with postoperative complications in hypospadias scoring systems [20, 22]. In the present cohort, however, clinically relevant curvature showed no statistically significant imbalance between TIP and GTIP and was corrected before urethroplasty. Thus, curvature-related severity was unlikely to represent a major measured source of imbalance.

By contrast, GTIP selection was driven by urethral plate morphology and intraoperative tubularization feasibility. Previous studies suggest that plate width, groove depth, and tissue quality may influence fistula formation and meatal stenosis, although width alone is an inconsistent predictor [6–9]. Therefore, our cohort should be interpreted as a urethral-plate-guided grafting cohort rather than a comparison of anatomically equivalent treatment groups.

### Positioning of our series and functional rationale

Our series reflects a pragmatic urethral-plate-guided strategy. GTIP was reserved for plates judged narrow, atrophic, or not safely tubularizable over an 8 Ch catheter without deep incision into the corpus spongiosum. TIP remained the default technique, and the relative use of the techniques and the indication strategy did not materially change over the study period. This differs from routine grafting in unselected distal hypospadias and more closely resembles studies focusing on narrow or unfavorable urethral plates [8, 14, 16, 18, 23].

Mechanistically, dorsal inlay grafting may provide an epithelialized surface along the incised plate and reduce secondary scar contraction, particularly after deep plate incision or in compromised tissue [10]. However, clinical evidence does not consistently show superiority across endpoints. In our cohort, stenosis rates were comparable between TIP and GTIP (9.4% vs. 10.3%), and fistula rates were not increased after GTIP (12.8% vs. 20.6%). Given the wide confidence intervals and non-randomized selection, these findings support the feasibility of GTIP as a selective adjunct rather than comparative superiority.

### Follow-up and timing of complications

The overall complication rate of 34.2% is comparatively high for a cohort dominated by distal hypospadias. Potential contributors include the inclusion of midshaft/proximal penile phenotypes, the broad definition of postoperative complications, heterogeneous and partly extended follow-up, ascertainment of late events, the tertiary referral setting, and selection bias introduced by requiring at least 6 months of follow-up. These factors cannot be quantified retrospectively, and the revision rate of 29.5% indicates that the elevated rate was not attributable solely to minor events. Complications were not confined to the early postoperative period: 10 of 29 patients remaining in follow-up for at least 60 months had a complication recorded after 60 months. This proportion is descriptive and may be inflated by outcome-dependent follow-up; it is not a cumulative incidence estimate. Nevertheless, the occurrence of late complications supports extended, developmentally structured follow-up, ideally including reassessment through adolescence and puberty, although the present cohort did not provide systematic puberty-spanning follow-up [24, 25].

### Strength and limitations

A major strength of this study is its consistent single-center intraoperative decision strategy, applied by a limited number of experienced pediatric urologists. The cohort reflects real-world decision-making in distal to proximal penile hypospadias and includes documentation of clinically relevant ventral curvature, which showed no statistically significant imbalance between groups.

Limitations include the retrospective design, non-randomized allocation, markedly unequal group sizes, and potential confounding by indication. GTIP was deliberately reserved for unfavorable urethral plates, meaning that the groups were not anatomically equivalent. The small GTIP cohort and wide confidence intervals limit precision; absence of statistical significance must not be interpreted as equivalence, and a type II error cannot be excluded.

Although meatal location and the presence of clinically relevant ventral curvature requiring correction were documented, urethral-plate-specific variables such as groove depth, elasticity, spongiosal quality, glans configuration, and objective plate width were not systematically quantified. The decision to perform GTIP therefore reflected pragmatic surgical judgment rather than a standardized anatomical score [7–9]. The exact method of angular quantification and individual curvature degrees were not consistently documented; the curvature variable represents clinically relevant curvature requiring correction according to the institutional threshold. Further limitations include possible follow-up bias from excluding patients with follow-up < 6 months, the absence of standardized postoperative photographs, the lack of blinded or independent outcome adjudication, the absence of routine uroflowmetry, and lack of standardized cosmetic or functional scores such as Hypospadias Objective Scoring Evaluation (HOSE) and Hypospadias Objective Penile Evaluation (HOPE) scores [26–28]. Consequently, the study compares documented complications and revisions but cannot establish functional or cosmetic equivalence or superiority.

### Clinical implications

Standard TIP remains the default technique for most distal and selected penile shaft repairs. GTIP should be considered an anatomy-guided modification for unfavorable urethral plate or tubularization conditions, not a competing standard procedure. Our findings support selective grafting but do not justify routine graft augmentation in unselected patients [13, 16, 18, 19, 23].

### Future directions

Future studies should prospectively separate anatomical severity domains, including meatal location, curvature, glans configuration, plate width, groove depth, and tissue quality. Structured intraoperative descriptors, validated cosmetic scores, uroflowmetry, and longer follow-up into adolescence are needed to refine indications for grafting and capture late functional outcomes [8, 23, 26–30].

## Conclusion

In this single-center cohort, selective GTIP in patients with unfavorable urethral plate morphology or conditions unsuitable for tension-free tubularization was associated with complication and revision rates closely aligned with those after standard TIP. Clinically relevant ventral curvature was not meaningfully imbalanced between groups and therefore seems unlikely to account for these findings. Although equivalence or superiority cannot be inferred from this study, the results support selective grafting as a clinically feasible component of a pragmatic intraoperative decision strategy.

## Supplementary Information

Below is the link to the electronic supplementary material.

Supplementary Material 1

## Data Availability

No datasets were generated or analysed during the current study.

## Declarations

### Use of AI

AI-assisted language editing (DeepL Translator) was used for linguistic refinement. All scientific content, data analysis, and conclusions were generated and verified by the authors.

## References

[1] Yu X, Nassar N, Mastroiacovo P et al (1980) Hypospadias prevalence and trends in international birth defect surveillance systems, 1980–2010. Eur Urol 76:482–490 10.1016/j.eururo.2019.06.027 PMC7265200 31300237

[2] Leunbach TL, Berglund A, Ernst A et al (2025) Prevalence, incidence, and age at diagnosis of boys with hypospadias: a nationwide population-based epidemiological study. J Urol 213:350–360 39626694 10.1097/JU.0000000000004319 PMC12708034

[3] Snodgrass W (1994) Tubularized, incised plate urethroplasty for distal hypospadias. J Urol 151:464–465 8283561 10.1016/s0022-5347(17)34991-1

[4] Snodgrass W, Koyle M, Manzoni G et al (1998) Tubularized incised plate hypospadias repair for proximal hypospadias. J Urol 159:2129–2131 9598557 10.1016/S0022-5347(01)63293-2

[5] Snodgrass WT, Bush N, Cost N (2010) Tubularized incised plate hypospadias repair for distal hypospadias. J Pediatr Urol 6:408–413 19837000 10.1016/j.jpurol.2009.09.010

[6] Holland AJ, Smith GH (2000) Effect of the depth and width of the urethral plate on tubularized incised plate urethroplasty. J Urol 164:489–491 10893631

[7] Nguyen MT, Snodgrass WT (2004) Effect of urethral plate characteristics on tubularized incised plate urethroplasty. J Urol 171:1260–1262 14767325 10.1097/01.ju.0000110426.32005.91

[8] Abbas TO, Braga LH, Spinoit AF et al (2021) Urethral plate quality assessment and its impact on hypospadias repair outcomes: a systematic review and quality assessment. J Pediatr Urol 17:316–325 33846072 10.1016/j.jpurol.2021.02.017

[9] Snodgrass W, Bush N (2014) Recent advances in understanding/management of hypospadias. F1000Prime Rep 6:101 25580255 10.12703/P6-101 PMC4229727

[10] Kolon TF, Gonzales ET (2000) The dorsal inlay graft for hypospadias repair. J Urol 163:1941–1943 10799234

[11] Gundeti M, Queteishat A, Desai D et al (2005) Use of an inner preputial free graft to extend the indications of Snodgrass hypospadias repair (Snodgraft). J Pediatr Urol 1:395–396 18947578 10.1016/j.jpurol.2005.03.010

[12] Ferro F, Vallasciani S, Borsellino A et al (2009) Snodgrass urethroplasty: grafting the incised plate—10 years later. J Urol 182:1730–1735 19692085 10.1016/j.juro.2009.03.066

[13] Silay MS, Sirin H, Tepeler A et al (2012) “Snodgraft” technique for the treatment of primary distal hypospadias: pushing the envelope. J Urol 188:938–942 22819401 10.1016/j.juro.2012.04.126

[14] Ahmed S, Noureldin YA, Sherif H et al (2021) Cosmetic outcomes of grafted tubularized incised plate urethroplasty in primary distal penile hypospadias: prospective comparative study with the classic Snodgrass repair. Afr J Urol 27:152

[15] Silay MS, Hoen ’T, Bhatt L et al (2021) Are there any benefits of using an inlay graft in the treatment of primary hypospadias in children? A systematic review and metanalysis. J Pediatr Urol 17:303–315 33691984 10.1016/j.jpurol.2021.02.013

[16] Helmy TE, Ghanem W, Orban H et al (2018) Does grafted tubularized incided plate improve the outcome after repair of primary distal hypospadias: a prospective randomized study? J Pediatr Surg 53:1461–1463 29680277 10.1016/j.jpedsurg.2018.03.019

[17] Mouravas V, Filippopoulos A, Sfoungaris D (2014) Urethral plate grafting improves the results of tubularized incised plate urethroplasty in primary hypospadias. J Pediatr Urol 10:463–468 24360521 10.1016/j.jpurol.2013.11.012

[18] Eldeeb M, Nagla S, Abou-Farha M et al (2020) Snodgrass vs snodgraft operation to repair the distal hypospadias in the narrow urethral plate. J Pediatr Urol 16:165.e1-165.e8 32144015 10.1016/j.jpurol.2020.01.006

[19] Alshafei A, Cascio S, Boland F et al (2020) Comparing the outcomes of tubularized incised plate urethroplasty and dorsal inlay graft urethroplasty in children with hypospadias: a systematic review and meta-analysis. J Pediatr Urol 16:154–161 32061491 10.1016/j.jpurol.2020.01.009

[20] Arlen AM, Kirsch AJ, Leong T et al (2015) Further analysis of the Glans-Urethral Meatus-Shaft (GMS) hypospadias score: correlation with postoperative complications. J Pediatr Urol 11:71.e1–5 25797855 10.1016/j.jpurol.2014.11.015

[21] Snodgrass W, Prieto J (2009) Straightening ventral curvature while preserving the urethral plate in proximal hypospadias repair. J Urol 182:1720–1725 19692004 10.1016/j.juro.2009.02.084

[22] Bandini M, Sekulovic S, Spiridonescu B et al (2020) Prevalence, assessment and surgical correction of penile curvature in hypospadias patients treated at one European Referral Center: description of the technique and surgical outcomes. World J Urol 38:2041–2048 31654219 10.1007/s00345-019-02961-x

[23] Taher MA, Wijaya NJ, Keane A et al (2025) Surgical repair techniques in hypospadias with unfavorable urethral plate: a systematic review and network meta-analysis. J Pediatr Urol 21:1109–1119 40000296 10.1016/j.jpurol.2025.02.009

[24] Faasse MA, Liu DB (2017) Early vs. late-presenting urethroplasty complications after hypospadias repair: a retrospective analysis of patient follow-up. J Pediatr Urol 13:354.e1-354.e5 28676152 10.1016/j.jpurol.2017.05.017

[25] Snodgrass W, Bush N (2022) Do new complications develop during puberty after childhood hypospadias repair. J Urol 208:696–701 35536670 10.1097/JU.0000000000002738

[26] González R, Ludwikowski BM (2011) Importance of urinary flow studies after hypospadias repair: a systematic review. Int J Urol 18:757–761 21883491 10.1111/j.1442-2042.2011.02839.x

[27] Rynja SP, de Jong TPVM, Bosch JLHR et al (2011) Functional, cosmetic and psychosexual results in adult men who underwent hypospadias correction in childhood. J Pediatr Urol 7:504–515 21429804 10.1016/j.jpurol.2011.02.008

[28] Van Der Toorn F, De Jong TPVM, De Gier RPE et al (2013) Introducing the HOPE (Hypospadias objective penile evaluation)-score: a validation study of an objective scoring system for evaluating cosmetic appearance in hypospadias patients. J Pediatr Urol 9:1006–1016 23491983 10.1016/j.jpurol.2013.01.015

[29] Bush NC, Villanueva C, Snodgrass W (2015) Glans size is an independent risk factor for urethroplasty complications after hypospadias repair. J Pediatr Urol 11:355.e1-355.e5 26320396 10.1016/j.jpurol.2015.05.029

[30] Bush NC, Snodgrass W (2017) Pre-incision urethral plate width does not impact short-term Tubularized Incised Plate urethroplasty outcomes. J Pediatr Urol 13:625.e1-625.e6 29133164 10.1016/j.jpurol.2017.05.020

