# Supplementary material for: Selective use of grafted TIP urethroplasty in distal to proximal penile hypospadias: a single-center cohort study
Source: World J Urol. 2026 Aug 21;44(1):594. doi: 10.1007/s00345-026-06690-w (PMC13498618; doi:10.1007/s00345-026-06690-w)
Supplement: Supplementary file 1 [file 345_2026_6690_MOESM1_ESM.docx]

**Supplementary Table 1. Follow-up summary**

| **Group** | **FU months mean ± SD** | **FU months median [IQR]** | **n ≥ 6m** | **n ≥ 12m** | **n ≥ 24m** | **n ≥ 5 years (60m)** |
| --- | --- | --- | --- | --- | --- | --- |
| Overall | 37.2 ± 28.1 | 26.6 [14.2–54.3] | 146 | 128 | 81 | 29 |
| TIP | 38.0 ± 29.5 | 26.4 [14.2–54.3] | 107 | 93 | 60 | 23 |
| GTIP | 35.2 ± 24.2 | 32.2 [13.8–53.2] | 39 | 35 | 21 | 6 |
| Distal | 38.3 ± 29.2 | 26.8 [14.1–59.1] | 115 | 101 | 64 | 25 |
| Mid+Prox penile | 33.2 ± 23.6 | 24.3 [14.2–46.7] | 31 | 27 | 17 | 4 |
| TIP – Distal | 39.2 ± 30.1 | 27.9 [14.8–59.3] | 90 | 78 | 52 | 21 |
| TIP – Mid+Prox penile | 31.5 ± 25.8 | 20.9 [13.1–43.9] | 17 | 15 | 8 | 2 |
| GTIP – Distal | 35.1 ± 26.1 | 23.9 [13.3–57.3] | 25 | 23 | 12 | 4 |
| GTIP – Mid+Prox penile | 35.3 ± 21.3 | 37.6 [15.2–48.3] | 14 | 12 | 9 | 2 |

**Footnote Supplementary Table 1:**
FU = follow-up; mo = months; SD = standard deviation; IQR = interquartile range. Data are presented as mean ± SD or median [IQR], as indicated. *n* denotes the total number of patients in each subgroup; *n* ≥ 6 mo, *n* ≥ 12 mo, *n* ≥ 24 mo, and *n* ≥ 60 mo denote patients with a minimum follow-up of 6, 12, 24, and 60 months, respectively.

**Supplementary Table 2. Occurrence of ≥1 complication during follow-up intervals**

| **Follow-up Interval** | **GTIP (n)** | **TIP (n)** | **Total (n)** |
| --- | --- | --- | --- |
| 0–24 months | 8 | 16 | 24 |
| 24–36 months | 2 | 5 | 7 |
| 36–48 months | 0 | 5 | 5 |
| 48–60 months | 1 | 4 | 5 |
| >60 months | 3 | 7 | 10 |

**Footnote Supplementary Table 2:**

Values represent the number of patients with at least one complication during the respective follow-up interval. Some patients experienced more than one complication and may therefore be represented in multiple intervals. The data are presented descriptively and do not reflect time-dependent incidence rates.

**Supplementary Table 3. Baseline distribution of clinically relevant curvature (≥20°)**

|  | **≥20° Curvature** | **<20° Curvature** | **Total** |
| --- | --- | --- | --- |
| GTIP | 7 (17.9%) | 32 (82.1%) | 39 |
| TIP | 22 (20.6%) | 85 (79.4%) | 107 |
| Column Total | 29 (19.9%) | 117 (80.1%) | 146 |
|  |  |  |  |

**Footnote Supplementary Table 3:**

Values are presented as n (%). Percentages are calculated per row. Curvature was assessed intraoperatively after degloving and artificial erection testing. Fisher’s exact test p = 0.82.

**Supplementary Table 4. Complication rates in patients with clinically relevant curvature ≥20°**

|  | **Complication** | **No Complication** | **Total** |
| --- | --- | --- | --- |
| GTIP | 0 (0.0%) | 7 (100%) | 7 |
| TIP | 7 (31.8%) | 15 (68.2%) | 22 |
| Column Total | 7 (24.1%) | 22 (75.9%) | 29 |

**Footnote Supplementary Table 4:**

Values are presented as n (%). Percentages are calculated per row. Fisher’s exact test p = 0.15.

**Supplementary Table 5. Complication rates in patients with curvature <20°**

|  | **Complication** | **No Complication** | **Total** |
| --- | --- | --- | --- |
| GTIP | 13 (40.6%) | 19 (59.4%) | 32 |
| TIP | 30 (35.3%) | 55 (64.7%) | 85 |
| Column Total | 43 (36.8%) | 74 (63.2%) | 117 |

**Footnote Supplementary Table 5:**

Values are presented as n (%). Percentages are calculated per row. Fisher’s exact test p = 0.67.

**Supplementary Table 6. Comparison of randomized trials and prospective comparative studies evaluating TIP versus GTIP**

| Study | Design | n (TIP / GTIP) | Hypospadias type | Indication for GTIP | Follow-up (months) | Complications TIP (%) | Complications GTIP (%) | Key difference | Authors’ conclusion |
| --- | --- | --- | --- | --- | --- | --- | --- | --- | --- |
| Helmy [16] | RCT | 30 / 30 | Distal | None (no UP selection) | 12 (fixed) | 3.3 | 6.7 | Longer OT time (GTIP) | No benefit of routine grafting |
| Mouravas [17] | RCT | 23 / 24 | Glandular–proximal penile | None | 38 (mean) | 30 | 8 | ↓ stenosis with GTIP | GTIP reduces stenosis |
| Eldeeb  [18] | RCT | 30 / 30 | Distal | UP < 8 mm | 12–16 (16 median) | 6.7 | 6.7 | No difference, Longer OT time (GTIP) | Selective grafting shows no clear advantage |
| Ahmed  [14] | Randomized comparative | 53 / 54 | Distal | None | 6 (fixed) | 9.5 | 9.3 | Longer OT time (GTIP) | Comparable outcomes |

**Footnote Supplementary Table 6:**

RCT = randomized controlled trial; UP = urethral plate; OT = operative time. Follow-up is reported as stated in the original publications (mean, median, or fixed duration). Complication rates are reported as percentages.

**Supplementary Table 7. Number of complications per patient** (N = 146, FU ≥ 6 months)

| Number of complications | n children | % |
| --- | --- | --- |
| 0 | 96 | 65.8 |
| 1 | 43 | 29.5 |
| 2 | 7 | 4.8 |
| ≥3 | 0 | 0 |

**Footnote Supplementary Table 7:**

Values represent the number of children experiencing 0, 1, 2 or ≥3 complications during minimum follow-up of 6 months; percentages refer to the total study population (N = 146).

**Supplementary Table 8. Total number of complication events**

| Complication type | n events | % of total events |
| --- | --- | --- |
| Meatal stenosis | 14 | 24.6 |
| Fistula | 27 | 47.4 |
| Glans dehiscence | 8 | 14.0 |
| Other | 8 | 14.0 |
| Total | 57 | 100 |

**Footnote Supplementary Table 8:**

Percentages refer to the total number of complication events (n = 57); multiple events could occur in a single patient.

**Supplementary Table 9. Operated vs. non-operated complications**

| Complication type | Total events | Operated (n, %) | Non-operated (n, %) |
| --- | --- | --- | --- |
| Fistula | 27 | 27 (100 %) | 0 (0 %) |
| Glans dehiscence | 8 | 3 (37.5 %) | 5 (62.5 %) |
| Meatal stenosis | 14 | 14 (100 %) | 0 (0 %) |
| Other | 8 | 5 (62.5 %) | 3 (37.5 %) |
| Total | 57 | 49 (86.0 %) | 8 (14.0 %) |

**Footnote Supplementary Table 9:**
Data are presented as n (%). Percentages refer to the total number of complication events per category. “Operated” indicates complications requiring surgical intervention, whereas “non-operated” indicates complications managed conservatively.
